# Spresso: an ultrafast compound pre-screening method based on compound decomposition

**DOI:** 10.1093/bioinformatics/btx178

**Published:** 2017-03-30

**Authors:** Keisuke Yanagisawa, Shunta Komine, Shogo D Suzuki, Masahito Ohue, Takashi Ishida, Yutaka Akiyama

**Affiliations:** 1 Department of Computer Science, School of Computing, Tokyo Institute of Technology, Ookayama, Meguro-ku, Tokyo, Japan; 2 Education Academy of Computational Life Sciences (ACLS), Tokyo Institute of Technology, Yokohama City, Kanagawa, Japan; 3 Department of Computer Science, Graduate School of Information Science and Engineering, Tokyo Institute of Technology, Ookayama, Meguro-ku, Tokyo, Japan; 4 Advanced Computational Drug Discovery Unit (ACDD), Institute of Innovative Research, Tokyo Institute of Technology, Yokohama City, Kanagawa, Japan

## Abstract

**Motivation:**

Recently, the number of available protein tertiary structures and compounds has increased. However, structure-based virtual screening is computationally expensive owing to docking simulations. Thus, methods that filter out obviously unnecessary compounds prior to computationally expensive docking simulations have been proposed. However, the calculation speed of these methods is not fast enough to evaluate ≥ 10 million compounds.

**Results:**

In this article, we propose a novel, docking-based pre-screening protocol named Spresso (Speedy PRE-Screening method with Segmented cOmpounds). Partial structures (fragments) are common among many compounds; therefore, the number of fragment variations needed for evaluation is smaller than that of compounds. Our method increases calculation speeds by ∼200-fold compared to conventional methods.

**Availability and Implementation:**

Spresso is written in C ++ and Python, and is available as an open-source code (http://www.bi.cs.titech.ac.jp/spresso/) under the GPLv3 license.

**Supplementary information:**

Supplementary data are available at *Bioinformatics* online.

## 1 Introduction

Protein tertiary structures and compounds contain essential information for drug discovery, and the availability of this information has increased in recent years. For example, the Protein Data Bank (PDB), which is the most popular public database of protein structures, contains > 114 000 entries, a 20% increase from 2014 to 2015 (Rose *et al.*, 2015). Moreover, the ZINC database of commercially available compounds contains ∼34 000 000 compounds (Irwin *et al.*, 2012). In drug discovery, the first step is to identify potential drug compounds specific to the target, followed by an optimization step to identify more feasible structures from among the potential drug compounds. Thus, the identification of potential drug compounds is therefore similar to ‘finding needles in a haystack’ (Klon *et al.*, 2004); thus, estimation of the likelihood for a compound to become a viable drug is critical in enhancing the effectiveness of searches. To estimate drug likelihood, computational methods called ‘virtual screening’ have been improved by the largeness of available databases (Sliwoski *et al.*, 2014). Furthermore, Chiba *et al.* (2015) pointed out that using multiple virtual screening methods results in obtaining viable drugs more efficiently based on the results of a potential drug identification contest.

Structure-based virtual screening (SBVS) is currently a standard step preceding wet-lab experiments during drug discovery (Cheng *et al.*, 2012). In SBVS, protein-ligand docking simulations are performed to estimate binding affinities (Meng *et al.*, 2011) and plausible binding modes for many drug candidates; however, this process is computationally demanding (Drwal and Griffith, 2013) because docking simulation is an optimization problem with many search degrees. The internal degree of freedom of a compound is a significant factor associated with the search space degrees and computational time required for docking simulations. For example, AutoDock Vina (Trott and Olson, 2010) spends ∼500 CPU seconds per compound, whereas the commercial docking tool Glide (Friesner *et al.*, 2004) is 50-fold faster than AutoDock Vina; however, its use is still not feasible to evaluate all available compounds in the ZINC database because of the time and cost involved. Given these limitations, studies have focused on screening compounds prior to docking, termed ‘pre-screening’ (Kumar and Zhang, 2015). These methods can be divided into two broad categories: ligand-based and structure-based (Drwal and Griffith, 2013; Ferreira *et al.*, 2015). Ligand-based approaches utilize known active/inactive compounds to screen candidate compounds, using machine learning methods or rule-based selection (Ripphausen *et al.*, 2011). These approaches are widely used as filtering methods and can deal with vast numbers of compounds, since ligand-based approaches are computationally less expensive than structure-based approaches. However, prediction based on known active/inactive compounds can lead to bias (Drwal and Griffith, 2013), and this method has difficulty finding drug candidates with different scaffolds from known active compounds. Structural docking-based methods avoid this problem, but require large computation times. For example, the high-throughput virtual-screening mode of Glide (Glide HTVS; Friesner *et al.*, 2004) and Panther (Niinivehmas *et al.*, 2015) can evaluate compounds up to 10-fold faster than ordinal docking tools, using a rough evaluation of affinity between ligand and protein. In particular, Glide HTVS has been widely used in recent studies for pre-screening (Mirza *et al.*, 2016; Muralidharan *et al.*, 2015). Nevertheless, the limited speed associated with this method precludes evaluation of all compounds available for purchase from ZINC in a reasonable computation time.

For these reasons, a much faster docking-based method sufficient to evaluate all ZINC compounds or any other compound libraries is urgently needed, despite its limited screening accuracy. In addition, it is not necessary for pre-screening methods to output structural conformation information because pre-screened candidates will subsequently undergo more expensive docking simulations.

To decrease computational cost, fragment-based methods have been adopted to calculate compound properties. For instance, topological polar surface area (TPSA; Ertl *et al.*, 2000) is a molecular polar surface area (PSA) estimation method that sums the fragment contributions and there is also a compound volume estimation method by counting each type of atom (Zhao *et al.*, 2003). Since docking score depends on proteins as well as compounds, docking score calculations by the fragment-based method is more difficult than that with compound properties; however, both property estimation methods suffice in terms of computational expenditure.

To address these concerns, we present a structure-based pre-screening method called Spresso (Speedy PRE-Screening method with Segmented cOmpounds, pronounced like ‘espresso’) that decomposes all candidate compounds into fragments with no internal degrees of freedom. These fragments are docked into target proteins, and compounds are roughly scored based on the results of fragment docking. Spresso performs ultrafast compound evaluation without protein-ligand conformation prediction. It utilizes the concept of compound decomposition from a previous docking program (eHiTS; Zsoldos *et al.*, 2007) and expands the concept by allowing reuse of fragment-docking results for analysis of different target compounds sharing the same fragment to enable ultrafast calculations in total.

## 2 Materials and methods

### 2.1 Elements of Spresso

**Fig. 1:**
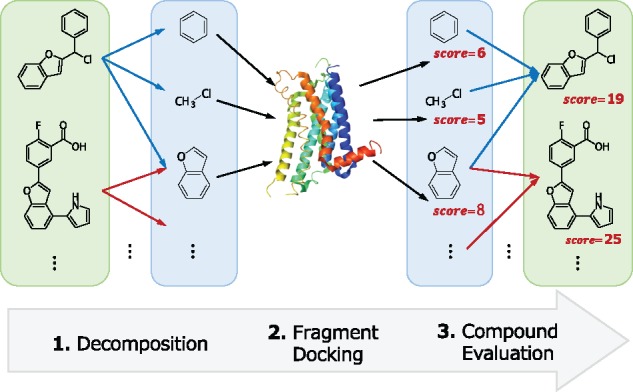
Spresso flowchart

**Fig. 2:**
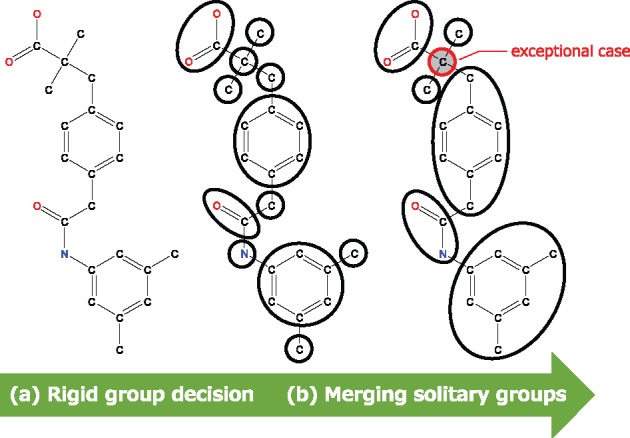
An example of compound decomposition. The carbon moiety in the structure on the right has four adjacent groups; therefore, it is not merged into any adjacent groups

The procedure of Spresso is comprised of three key steps summarized in Figure 1: (i) compound decomposition (Fig. 2), (ii) fragment docking and (iii) fragment-based evaluation of each compound score.

#### 2.1.1 Compound decomposition

Several compound decomposition rules have been proposed, with the most famous being RECAP (REtrosynthetic Combinatorial Analysis Procedure; Lewell *et al.*, 1998). RECAP was originally created for combinatorial chemistry, thus a compound will be fragmented in a restrictive manner. Generally, the smaller the internal degree of freedom of a structure, the faster the docking calculation speed. Therefore, more bonds should be cleaved by Spresso than that by RECAP to accelerate the process. Thus our decomposition strategy creates rigid fragments with no internal degree of freedom. In our method, a fragment is defined as a rigid substructure without considering hydrogen atoms because they are sometimes ignored in docking calculations. To divide compounds into appropriate fragments, a two-step algorithm is used. (i) The first step involves rigid-group determination: all ring systems are considered rigid, even in the case of cyclohexane. Acyclic fragments with double, triple, or resonance bonds and sp2-hybridized atoms are also considered rigid. (ii) The second step involves merging solitary groups (single-atom fragments): each non-solitary group and its adjacent solitary group are merged, except for solitary groups having three or more adjacent groups. Figure 2 shows an example of ligand decomposition. As exceptional cases, single atom fragments can exist in some compounds. Whenever a bond is broken during this decomposition, hydrogen atoms are added.

Another benefit of decomposition is the sharing of docking results for duplicated fragments. Generally, there are many derivatives in a compound library, resulting in vast numbers of duplicate fragments. This duplication allows us to reduce the total number of necessary fragment docking simulations. The effect of decomposition from compound to fragment depends upon the compound library and the degree of decomposition, since increasing the number of pre-screened compounds and cleaved compound bonds will accelerate subsequent fragment docking compared to compound docking without decomposition.

#### 2.1.2 Fragment docking

After decomposition, all rigid fragments are docked to the best location regardless of the other fragments. This means that all fragments are independently docked to the location in the protein cavity where they fit best. Fragments having the same substructures as those from different compounds can be scored identically, thereby significantly decreasing the number of fragments needing to be docked. The best score from the docking results for each fragment is recorded. For this procedure, any docking tool capable of outputting a score can be used, including AutoDock Vina (Trott and Olson, 2010), Glide (Friesner *et al.*, 2004), or GOLD (Verdonk *et al.*, 2003).

#### 2.1.3 Fragment-based evaluation of each compound

Compounds are evaluated after fragment docking is completed. Given that only fragments are docked into the target protein, we cannot obtain docking scores for entire compounds. Therefore, the screening evaluation score for each compound must be calculated based on the docking scores of fragments decomposed from the original compound. There are two strategies for compound evaluation: (i) choosing combinations of fragment conformations that avoid contradictions, and (ii) choosing the best conformation without consideration of fragment collisions. The former strategy is more precise as compared to the latter strategy, but searching for conformation combinations can also be computationally expensive. Given our goal of creating a computationally faster ‘pre-screening’ method, we chose the latter strategy for compound evaluation.

We can consider many formulae for calculating compound-evaluation scores from fragment-docking scores (${score}_{f}$). In this study, we evaluated seven calculation formulae. 

(I) Summation of fragment-docking scores (SUM)

(1) $$SUM=\sum_{f}score_{f}$$

Summation is one of the simplest evaluation methods, where SUM reflects the approximate rough upper bound of the compound-docking score. Generally, the SUM value is larger when a compound is divided into more fragments.

(II) Best value of fragment-docking scores (MAX)

(2) $$MAX=\underset{}{\max}score_{f}$$

Utilizing the best value is also a simple evaluation method, where MAX reflects the estimated rough lower bound of compound-docking scores. In most cases, the MAX value will be less than the compound-docking score; however, a docking score associated with a single fragment may exceed the compound-docking score in specific cases (i.e. a compound too large for a protein cavity).

(III) Generalized Sum (GS)

(3) $${GS}_{x}=\sqrt[{x}]{\sum_{f}{\left(score_{f}\right)}^{x}}$$

GS_1_ is equal to SUM, while GS_∞_ is equal to MAX; therefore, GS can express the mixture of SUM and MAX values continuously. In this study, we chose (III) GS_3_ from the GS_2_∼GS_10_ evaluation results (Supplementary Fig. S1). 

The other four calculation formulae are shown in supplementary text S1. GS requires non-negative values as input, while the fragment-docking score is almost always a negative value because the score was fitted to experimental ΔG. Therefore, the fragment-docking scores are inverted, and positive docking scores (which are inverted to negative values) are treated as zero.

The best pre-screening accuracy was achieved when (III) GS_3_ was used (detailed description provided in Section 3.2); thus, GS_3_ was adopted as the default formula in Spresso.

### 2.2 Datasets

The Directory of Useful Decoys, Enhanced (DUD-E; Mysinger *et al.*, 2012) was used to evaluate the performance of pre-screening during the virtual-screening process. The DUD-E dataset is widely used and consists of 102 diverse sets of protein targets, as well as active and decoy compounds. The ZINC database (Irwin *et al.*, 2012) was also used to measure calculation time, since the number of active compounds and decoys in each set is insufficient as compared to those used in actual virtual screening. We chose ‘all purchasable’ and ‘all boutique’ datasets, then eliminated duplication based on ZINC ID. The total number of compounds was 28 629 602.

### 2.3 Implementation

The code for fragment decomposition was written in C ++, and the compound-evaluation score calculations were written in Python. Spresso code is freely available at http://www.bi.cs.titech.ac.jp/spresso/ under the GPL version 3 license. We used Glide SP mode and Glide HTVS mode for fragment docking, and used Glide HTVS to dock compounds for comparison.

### 2.4 Computing environment

All calculations were conducted on the TSUBAME 2.5 supercomputing system, Tokyo Institute of Technology, Japan. We used its thin nodes in all experiments, with each node having two Intel Xeon X5670 CPUs (six cores/CPU) and 54 GB of RAM. Because Glide software is a single-thread program, all docking simulations were performed in parallel using 12 CPU cores. It should be noted that Glide is a proprietary software, and thus it cannot be optimized for specific computing environments.

### 2.5 Metrics

Two computational experiments were conducted: (i) evaluation of calculation speed, and (ii) evaluation of virtual screening accuracy. Since one license will allow us to use only one CPU core, we used CPU time to evaluate calculation speed. Accuracy was measured by performance efficiency according to enrichment factors (EFs) (Hamza *et al.*, 2012).

(4) $${EF}_{x\%}=\frac{Pos_{x\%}/All_{x\%}}{Pos_{100\%}/All_{100\%}}$$

In Eq. 4, ${Pos}_{x\%}$, ${All}_{x\%}$, ${Pos}_{100\%}$ and ${All}_{100\%}$ are the number of active compounds in the top *x%* of screened compounds, the number of compounds in the top *x%* of screened compounds, the total number of screened active compounds, and the total number of screened compounds, respectively. In virtual screening, it is pragmatically meaningless to assess differences between lower ranked compounds because wet-lab experiments can be executed up to only a few thousand compounds even though computational methods can deal with more than 1 million compounds. Therefore, ${EF}_{1\%}$ and ${EF}_{2\%}$ were calculated to evaluate accuracy.

### 2.6 Assessment of prediction accuracy

As previously mentioned, Spresso is not intended for independent use. Therefore, an evaluation must involve not only Spresso but also a following compound docking calculation. The procedure used for evaluation of accuracy was as follows: (i) with each pre-screening method, 2%, 5%, or 10% of the number of all target compounds were selected; (ii) pre-screened candidates were docked using Glide SP to obtain a docking score; and (iii) the top 1% and 2% of compounds were used to calculate ${EF}_{1\%}$ and ${EF}_{2\%}$. We calculated five combinations for each pre-screening method.

## 3 Results

To evaluate the usefulness of Spresso with regard to speed and prediction accuracy, two experiments were performed. In all experiments, Glide HTVS, which is a conventional pre-screening method, was also evaluated for comparison.

### 3.1 Comparison of docking-calculation speed

Table 1 shows the calculation times for docking of all 28 629 602 ZINC compounds into three target proteins from the DUD-E dataset. Spresso using Glide SP-mode fragment docking (Spresso-SP) required < 2 CPU days, and Spresso with Glide HTVS-mode fragment docking (Spresso-HTVS) required < 1 CPU day, while whole-compound docking using Glide HTVS mode required > 4 CPU months. These results suggest that Spresso is up to ∼200-fold faster than compound docking with conventional Glide HTVS pre-screening.

**Table 1:** The results of docking times for docking of all 28 629 602 ZINC compounds into three DUD-E protein targets

Target	Calculation time [CPU hours]		
	Spresso-SP	Spresso-HTVS	Glide HTVS
ACES	42.6 (× 76.8)	22.8 (× 143.1)	3268.8
EGFR	38.9 (× 126.4)	21.5 (× 229.3)	4925.1
PGH1	41.8 (× 88.0)	20.9 (× 175.4)	3674.5

Values in parentheses indicate the fold increase in speed exhibited by Spresso relative to Glide HTVS.

### 3.2 Prediction accuracy in DUD-E benchmarking

**Fig. 3:**
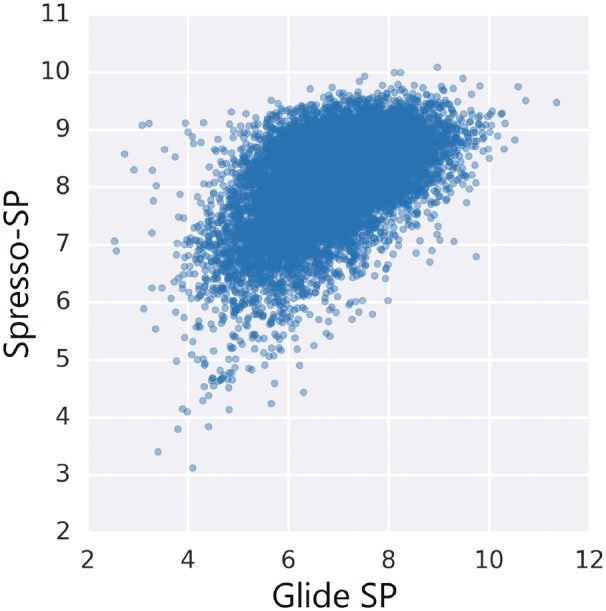
A scatter plot of the Glide SP score and the Spresso-SP score for DUD-E CP3A4 target. Each dot represents a compound in DUD-E CP3A4 dataset. The correlation coefficient is R = 0.55

Table 2 shows the average EF values associated with each DUD-E target. The formulae for SUM, MAX and GS_3_ in Table 2 are listed as (I)–(III) in Section 2.1, respectively. The other four calculation formulae were also evaluated (Supplementary Table S1). Eight score calculations were evaluated, revealing that the combination of Spresso-SP and GS_3_ was the best. Spresso-HTVS exhibited slightly less accurate results as compared to Spresso-SP. The superiority of Spresso-SP was more obvious with GS. These differences were dependent upon the docking tools used. Our results indicate that Spresso was less accurate when compared with conventional method. Pearson’s correlation with Glide SP score also showed Spresso-SP (GS_3_) is slightly less similar to (R = 0.55, Fig. 3) than Glide HTVS (R = 0.60, Supplementary Fig. S2) for CP3A4, one of the DUD-E target. These results represent a major disadvantage of Spresso; however, we believe this loss in accuracy can be compromised owing to the method’s unprecedented calculation speed based on the fact that the previous method (Glide HTVS) requires impermissibly longer computation time.

**Table 2:** The results of averaged prediction accuracy for 102 DUD-E targets

Methods		Enrichment factors				
		2%–1%	5%–1%	10%–1%	5%–2%	10%–2%
Spresso-SP	SUM	4.58	6.78	8.92	4.00	5.53
	MAX	9.28	11.01	11.94	7.51	8.31
	GS_3_	**9.73**	**12.79**	**15.03**	**8.01**	**9.94**
Spresso-HTVS	SUM	4.60	6.78	8.93	4.20	5.46
	MAX	9.29	9.93	12.41	6.38	8.29
	GS_3_	9.00	12.18	14.49	7.39	9.24
Glide HTVS		17.85	18.97	19.60	12.50	12.92

Note: All enrichment factors represent the average of 102 EFs from DUD-E protein targets. (a%–b%) indicates the EF_b%_ when compounds were prescreened using a% of all compounds. Best EF values among Spressos are written in bold.

**Fig. 4:**
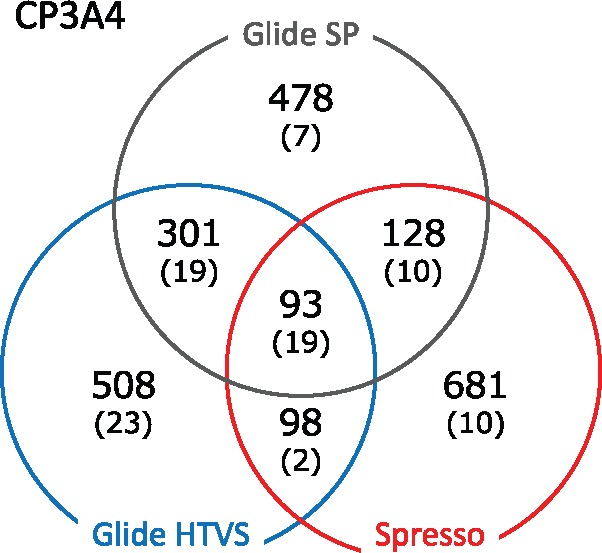
A Venn diagram of selected compounds identified by pre-screening for CP3A4, a DUD-E target. The top 1000 compounds identified by Glide SP, Glide HTVS and Spresso-SP are shown. The number of compounds for each method is shown and the numbers of true positives are in parentheses

In order to reveal how many compounds selected by Glide SP are included in the compounds selected by pre-screening methods Glide HTVS or Spresso, the overlap in selected compounds identified with each method was calculated for DUD-E Diverse Subset (8 targets). Venn diagrams are shown in Figure 4 and Supplementary Figures S3–S9. These diagrams indicate that the compounds identified with Spresso have less intersection with those from Glide SP than Glide HTVS.

## 4 Discussion

### 4.1 Number of unique fragments

The primary reason for the accelerated docking speeds observed was the reduction in number of docking trials. In this case, 28 629 602 compounds in the ZINC database were decomposed into 263 319 fragments; thus, the number of fragments was approximately one-hundredth of the number of compounds, resulting in ∼100-fold decreases in docking time. The number of fragments is dependent on the database; for example, the ChEMBL version 21 database (Bento *et al.*, 2014) contains 1 583 897 compounds, which were decomposed into 127 360 fragments (a ∼10-fold reduction). As for the PubChem (Kim *et al.*, 2016) compound database, 88 527 810 compounds have a molecular weight < 1000 Da, and they were decomposed into 2 082 185 fragments (a ∼40-fold reduction). Additionally, the RECAP rule decomposed 28 629 602 ZINC compounds into 3 161 753 fragments, approximately 12-times larger than that by our decomposition rule. For this reason, RECAP is unfeasible for use in our pre-screening method.

### 4.2 Superiority of $GS_{3}$

The computational experiment in section 3.2 revealed the (III) GS_3_ formula as the best of the eight possible methods for calculating compound-evaluation score. Method (I) SUM, utilizing all fragment scores equally, was the worst of the eight, while GS_*x*_ returned acceptable results. The GS_*x*_ exponent acts as a weight coefficient, which implies that the result indicates that higher-scoring fragments should be more weighted. However, GS_3_ returned more accurate results relative to method (II) MAX, given that considering the top few fragment scores is more informative than considering only the top fragment score.

### 4.3 Score fitting to Glide SP

Linear least squares fitting is often applied to experimental results or precise estimates in fragment-based, compound property estimation methods. In the compound property estimation methods, common explanatory variables include the fragment type, number of cleaved bonds and number of rings, amongst others; however, it is inappropriate to determine the contribution of each fragment in docking simulations since docking scores differ based on the target protein, and thus fragment-docking scores are used with equal contribution. Additionally, the number of cleaved bonds must affect the sum of fragment score. Because of above reasons, we generated a linear regression model with two factors, ${score}_{SUM}$ and the number of cleaved bonds, performed fittings with the Glide SP compound docking score as a target using the DUD-E HIVPR dataset, and then calculated the DUD-E CP3A4 dataset compounds’ ${score}_{fitting}$ with the fitted parameter. The data utilized for this pre-screening is detailed in Supplementary Table S2. The correlation coefficient between ${score}_{fitting}$ and Glide SP of CP3A4 was R = 0.49 (Supplementary Fig. S10), which is lower than that between GS_3_ and Glide SP (R = 0.55, Fig. 3), thus the linear regression fitting did not work well and explanatory valiables should be more considered.

### 4.4 Can Spresso conserve compound diversity?

Drwal and Griffith (2013) showed that structure-based methods are likely to maintain the diversity of compound structures as compared with ligand-based methods. While this is one reason to use structure-based methods, it does not guarantee that the diversity of compounds selected by Spresso will be maintained. We analyzed the diversity of compounds selected by Spresso according to two characteristics: physicochemical features and structural diversity. We focused on three DUD-E targets (PGH1, ACES and EGFR) and screened ZINC compounds using Spresso-SP, Glide HTVS and a ligand-based method. As for the ligand-based method, a support vector machine (SVM) with RBF kernel was adopted because it is one of the most popular machine learning methods for ligand-based screening. ECFP4 fingerprint (Rogers and Hahn, 2010) was used for input feature vectors of SVM. The details associated with the SVM are shown in Supplementary Table S3. The logP and the molecular weight of the top 0.1% of compounds were calculated in order to assess the bias of physicochemical features. Additionally, the maximum Tanimoto coefficient value between each known active compound was also calculated based on ECFP4 fingerprint in order to assess structural diversity. A high Tanimoto coefficient between two compounds indicates that the two structures share structural similarity.

**Fig. 5:**
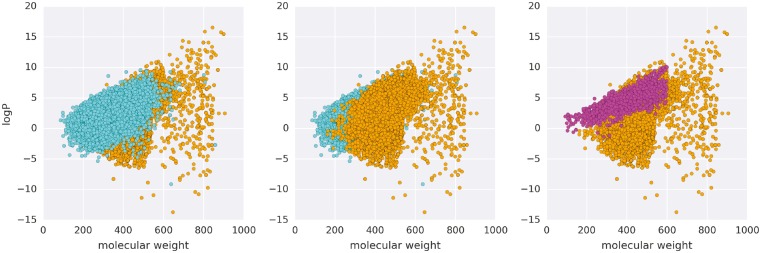
Scatter plot of physicochemical features based on pre-screening for ACES, a DUD-E protein target. Each dot represents a compound: cyan dots represent 0.1% of the compounds from the ZINC database; orange dots represent the top 0.1% of Spresso-SP compounds calculated using the method (III) GS_3_ formula; and magenta dots represent active compounds for ACES from the DUD-E dataset

**Fig. 6:**
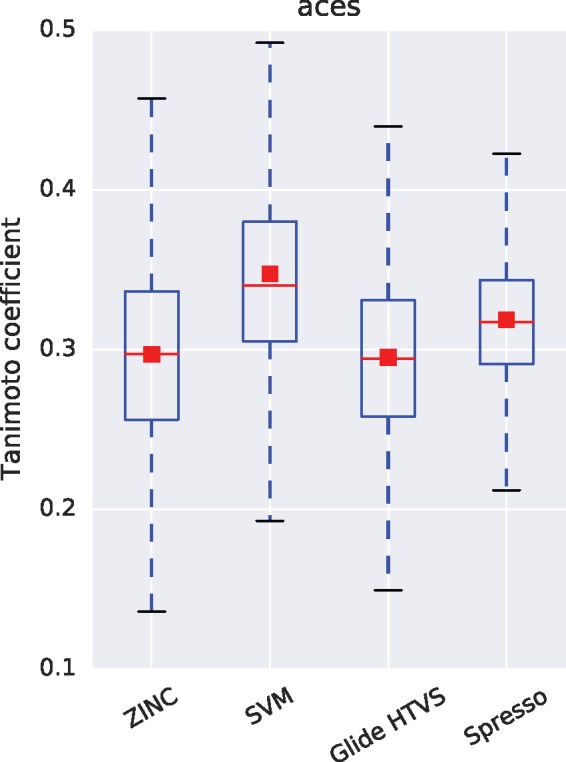
Boxplot representation and average (square dots) of the maximum Tanimoto coefficient between active compounds of target ACES. The data indicate structural diversity. ZINC, SVM, Glide HTVS and Spresso represent 0.1% of randomly selected compounds from the ZINC database, the top 0.1% of compounds resulting from SVM prediction, the top 0.1% of compounds resulting from Glide HTVS scoring, and the top 0.1% of compounds returned from Spresso-SP results using method (III) GS_3_ scoring, respectively

Results of these assessments for the ACES target are shown in Figures 5 and 6. Figure 5 shows that Spresso is likely to assign higher scores to large compounds. This is expected in some cases, because larger compounds are more likely to obtain higher scores in docking simulations (Verdonk *et al.*, 2004); however, compounds that are too large to enter protein cavities must be omitted. Figure 5 shows that Spresso conserved structural diversity on the same scale as that observed with Glide HTVS, while bias toward known active compounds was observed in results from the ligand-based method (SVM). Assessment results for EGFR and PGH1 are shown in Supplementary Figures S11–S14.

### 4.5 Large-compound cutoff value

According to our results, compounds with a larger volume than that of the target protein’s cavity should be omitted before Spresso pre-screening. We propose a cutoff method that compares compound volume and protein-cavity volume with the following provisions: (i) the volumes of all compounds ($V_{c}$) are estimated with the formula proposed by Zhao *et al.* (2003); (ii) the volume of the protein cavity ($V_{p}$) is estimated by Sitemap (Halgren, 2009); and (iii) compounds where $V_{c}$ exceeds $kV_{p}$ (*k* is a parameter) are omitted.

Parameter *k* represents the flexibility of the protein cavity, and *k* = 1 indicates that the volume of the protein cavity is used as the threshold of the compound volume; however, this is too strict because of protein flexibility. False negatives will occur when *k* is too small, while false positives will occur when *k* is too large. Here, we adopted *k* = 1.5 in order to restrict false negatives. This cutoff parameter moderately omitted compounds that were too large (Supplementary Figs S15–S17). This method does not require any information prior to setting the cutoff value, thereby eliminating structural bias (Supplementary Figs S18–S20).

However, a tendency for selecting larger compounds still remains a problem for Spresso, with smaller compounds likely to be eliminated. Therefore, the application of penalty parameters as part of the fragment score (e.g. fragment efficiency, which is similar to ligand efficiency (Shultz, 2013)) should be considered as future work in order to avoid bias in selecting feasible small compounds.

### 4.6 The top-screened compounds by Spresso

**Fig. 7:**
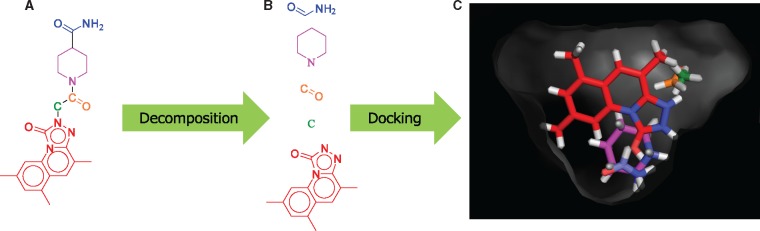
(**A**) Structure of ZINC12181222, the highest scoring compound for the protein target ACES. (**B**) Result of ZINC12181222 decomposition. (**C**) Results of fragment docking. The color of the structure mimics those of the structures shown in (A) and (B)

The highest scoring compound for the target protein ACES is shown in Figure 7. The top compound screened by Spresso-SP with a cutoff value was ZINC12181222 (Fig. 7A), with a molecular weight of 395.5 Da and a logP of 1.84. These physicochemical features indicate a likely drug compound according to Lipinski’s rule of five (Lipinski *et al.*, 1997). The decomposition and fragment-docking results are shown in Figure 7B and C. Since Spresso did not consider collisions between fragments in order to keep computation time low, some fragments appear to have collided (Fig. 7C). Interestingly, the best compound still exhibited a reasonable molecular weight according to Lipinski’s rule despite the collisions. Our findings indicate that the cutoff method was capable of omitting compounds unable to dock given target proteins.

## 5 Conclusion

In this study, we described Spresso, a docking-based pre-screening method for database-wide screening. In order to evaluate all compounds from large databases within a practical amount of time, Spresso uses compound decomposition into fragments, resulting in reuse of fragment scores, followed by fragment-docking results to estimate screening values without structure reconstruction. Our results showed that Spresso achieved up to ∼200-fold faster calculation using ∼29 million compounds as compared to compound docking by Glide HTVS. This acceleration rate is positively correlated to the number of compounds in a target database. Consequently, this method is capable of screening over tens of millions of compounds with limited computational resources.

For compound evaluation, the GS_3_ formula was adopted; however, according to the physicochemical assessment, Spresso-preferred compounds are likely to be large, despite the need to filter compounds too large for a given target protein cavity. We proposed a cutoff based on protein cavity volume, which requires further validation. Furthermore, future work should improve prediction accuracy (enrichment factors) by partially considering collisions between fragments, which may only slightly increase computation time, e.g. by 5-fold.

The computational efficiency of Spresso enables the screening of large compound databases within realistic times. In order to manage chemical compound libraries that continue to increase in size, corresponding increases in computational speed are necessary for virtual screening.

## Supplementary Material

Supplementary Data
