# Supplementary material for: Spresso: an ultrafast compound pre-screening method based on compound decomposition
Source: Bioinformatics. 2017 Mar 30;33(23):3836–43. doi: 10.1093/bioinformatics/btx178 (PMC5860314; doi:10.1093/bioinformatics/btx178)
Supplement: Supplementary Data [file btx178_final_version_suppl.docx]

*Supplementary Information*

Spresso: An ultrafast pre-screening method based on compound decomposition

Keisuke Yanagisawa^1,2^, Shunta Komine^2,3^, Shogo D. Suzuki^2,3^, Masahito Ohue^1,4^,
Takashi Ishida^1,2,4^, and Yutaka Akiyama^1,2,4*^

^1^Department of Computer Science, School of Computing, Tokyo Institute of Technology, W8-76 2-12-1, Ookayama, Meguro-ku, Tokyo 152-8550, Japan

^2^Education Academy of Computational Life Sciences (ACLS), Tokyo Institute of Technology, J3-141 4259, Nagatsutacho, Midori-ku, Yokohama City, Kanagawa 226-8501, Japan

^3^Department of Computer Science, Graduate School of Information Science and Engineering, Tokyo Institute of Technology, W8-76 2-12-1, Ookayama, Meguro-ku, Tokyo 152-8550, Japan

^4^Advanced Computational Drug Discovery Unit (ACDD), Institute of Innovative Research, Tokyo Institute of Technology, 4259 Nagatsutacho, Midori-ku, Yokohama City, Kanagawa 226-8501, Japan

^*^Corresponding author (akiyama@c.titech.ac.jp)

**Text S1. Fragment-based evaluation formulae**

As mentioned in the main text, we used seven formulae, four of which were not presented in the main article. These are based on the means of (I) SUM and (II) MAX.

1. Arithmetic Mean (AM)

$$AM=\frac{SUM+MAX}{2}$$

(S1)

1. Z-scored Arithmetic Mean (zAM)

$$zAM=\frac{zscore\left( \mathrm{SUM} \right)+zscore(\mathrm{MAX})}{2}$$

(S2)

If the means of two scores are calculated, the SUM value will have a greater effect on the final mean value than on the MAX value as the SUM has a wider range. Thus, a z-scored SUM value and z-scored MAX value are used in place of the raw SUM and MAX values to balance this influence.

1. Geological Mean (GM)

$$GM=\sqrt{MAX\cdot SUM}$$

(S3)

1. Harmonic Mean (HM)

$$HM=\frac{2\cdot MAX\cdot SUM}{MAX+SUM}$$

(S4)

The prediction accuracies for the four formulae and those of (I) SUM, (II) MAX, and (III) GS_3_ are shown in Table S1.

**Table S1.** Results of the averaged prediction accuracy for 102 DUD-E targets.

| Methods | | Enrichment Factors | | | | |
| --- | --- | --- | --- | --- | --- | --- |
|  |  | 2%–1% | 5%–1% | 10%–1% | 5%–2% | 10%–2% |
| Spresso-SP | SUM | 4.58 | 6.78 | 8.92 | 4.00 | 5.53 |
|  | MAX | 9.28 | 11.01 | 11.94 | 7.51 | 8.31 |
|  | GS_3_ | **9.73** | **12.79** | **15.03** | **8.01** | **9.94** |
|  | AM | 5.09 | 7.35 | 9.37 | 4.55 | 5.87 |
|  | zAM | 7.14 | 10.01 | 12.58 | 6.49 | 8.39 |
|  | GM | 6.56 | 9.23 | 11.04 | 5.75 | 7.18 |
|  | HM | 7.51 | 9.64 | 11.61 | 6.32 | 7.90 |
| Spresso-HTVS | SUM | 4.60 | 6.78 | 8.93 | 4.20 | 5.46 |
|  | MAX | 9.29 | 9.93 | 12.41 | 6.38 | 8.29 |
|  | GS_3_ | 9.00 | 12.18 | 14.49 | 7.39 | 9.24 |
|  | AM | 5.18 | 7.69 | 9.27 | 4.58 | 5.82 |
|  | zAM | 7.00 | 10.04 | 12.67 | 6.26 | 8.33 |
|  | GM | 6.78 | 9.15 | 10.87 | 5.64 | 6.94 |
|  | HM | 6.85 | 9.40 | 11.98 | 5.93 | 7.79 |
| Glide HTVS | | 17.85 | 18.97 | 19.60 | 12.50 | 12.92 |

*Note:* All enrichment factors (EFs) represent the average of 102 EFs from the DUD-E protein target. a%-b% indicates the EFb%, when compounds were pre-screened using a % of all compounds. The best EF values among Spresso methods are shown in bold.

**Fig. S1**. Results of the averaged prediction accuracy for 102 DUD-E targets. This figure shows 2%–1% results that represent the EF1% when 2% of all compounds were pre-screened, and suggests that GS_3_ is the best parameter.


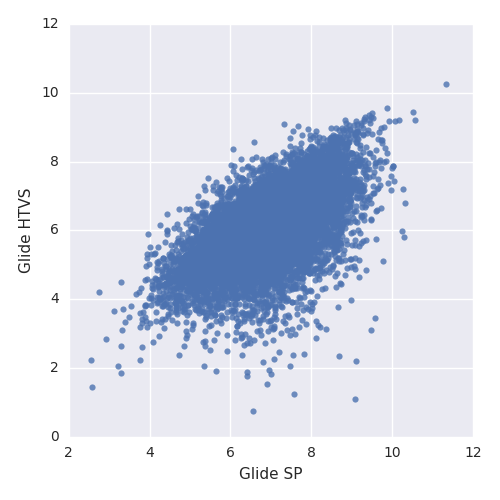


Glide HTVS (-kcal/mol)

Glide SP (-kcal/mol)

**Fig S2.** A scatter plot of the Glide SP and Glide HTVS scores for the DUD-E CP3A4 target. Each dot represents a compound in the DUD-E CP3A4 dataset. The correlation coefficient is R = 0.60.

| **** | **** |
| --- | --- |
| **** | **** |
| **** | **** |
| **** | |

**Fig. S3–S9.** Venn diagrams of selected compounds identified by pre-screening for 7 DUD-E targets. The top 1,000 compounds identified by Glide SP, Glide HTVS, and Spresso-SP are shown. The number of compounds for each method is shown, and numbers of true positives are in parentheses.

**Table S2.** Information used to generate the linear regression model and estimation.

| **Model creation** | Protein | DUD-E HIVPR (PDBID: 1XL2) |
| --- | --- | --- |
|  | Compound | Actives/Decoys of DUD-E HIVPR (36286 compounds) |
|  | Target score | The score of Glide SP compound docking |
|  | Base model | $\mathrm{score}_{\mathrm{fitting}}=a\cdot\mathrm{scor}e_{\mathrm{SUM}}+b\cdot\left\vert\#cleaved bonds \right\vert$ |
| **Score estimation** | Protein | DUD-E CP3A4 (PDBID: 3NXU) |
|  | Compound | Actives/Decoys of DUD-E CP3A4 (11970 compounds) |
| **Calculation Library** | | Python StatsModels |

**
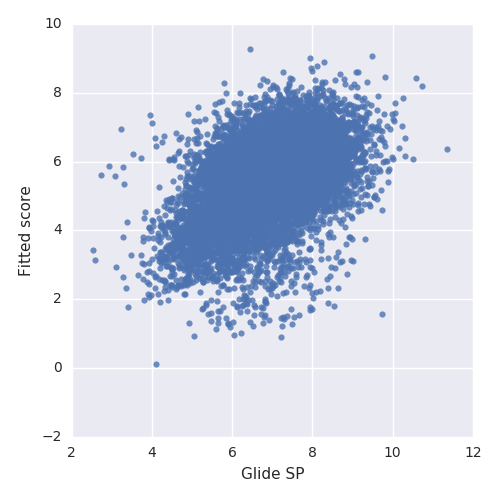
**

Glide SP (-kcal/mol)

Fitted score

**Fig S10.** A scatter plot of the Glide SP and fitted scores for the DUD-E CP3A4 target. Each dot represents a compound in the DUD-E CP3A4 dataset. The correlation coefficient is R = 0.49.

**Table S3.** Machine-learning settings on the support vector machine (SVM)^a^

| DUD-E Target name | **Training data** | | **Best parameter** | |
| --- | --- | --- | --- | --- |
|  | #Active compounds | #Inactive compounds | *C*: Cost parameter | *γ*: RBF-kernel parameter |
| ACES | 1,635 | 487 | 2^3^ | 2^−5^ |
| EGFR | 1,620 | 407 | 2^3^ | 2^−7^ |
| PGH1 | 543 | 1,070 | 2^3^ | 2^−5^ |

^a^Active compounds and decoys were obtained from the DUD-E dataset. We performed parameter tuning by adopting the optimum-cost parameter C and the RBF-kernel parameter γ from grid-search results during three-fold cross validation.


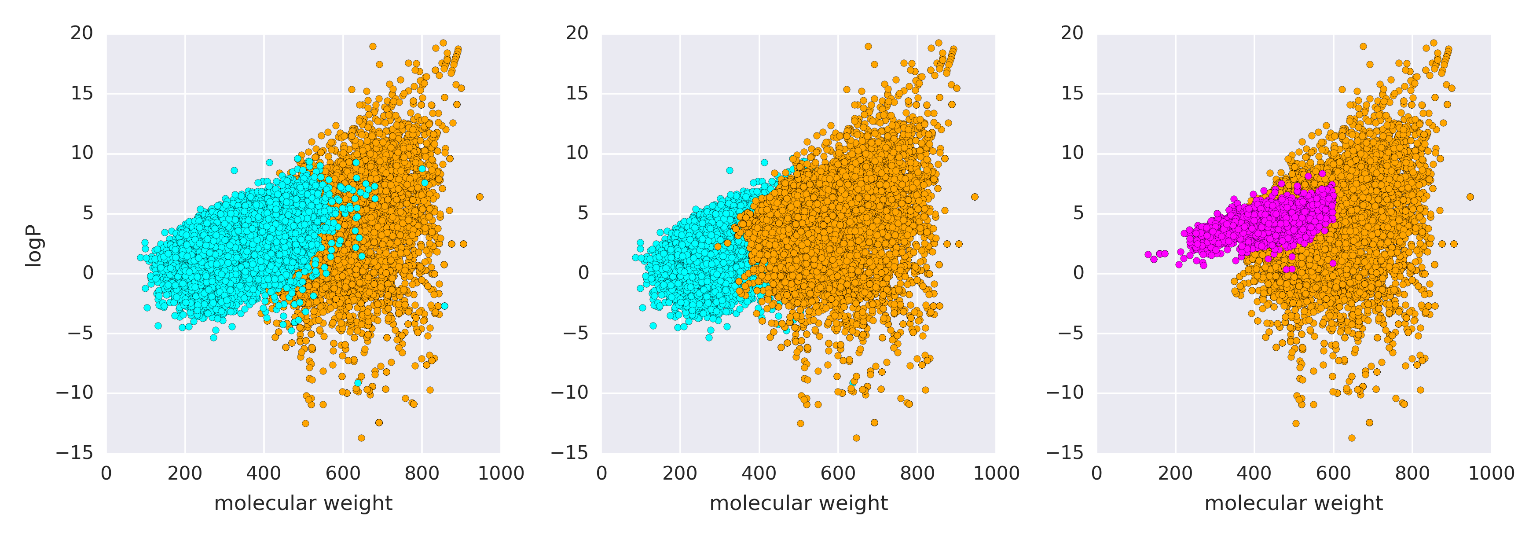
**Fig. S11.** The scatter plot of physicochemical features based on pre-screening for EGFR, a DUD-E target. Each dot represents a compound: cyan dots represent 0.1% of compounds from the ZINC database, orange dots represent the top 0.1% of Spresso-SP results using the method (III) GS_3_ formula, and magenta dots represent active compounds for EGFR from the DUD-E dataset.


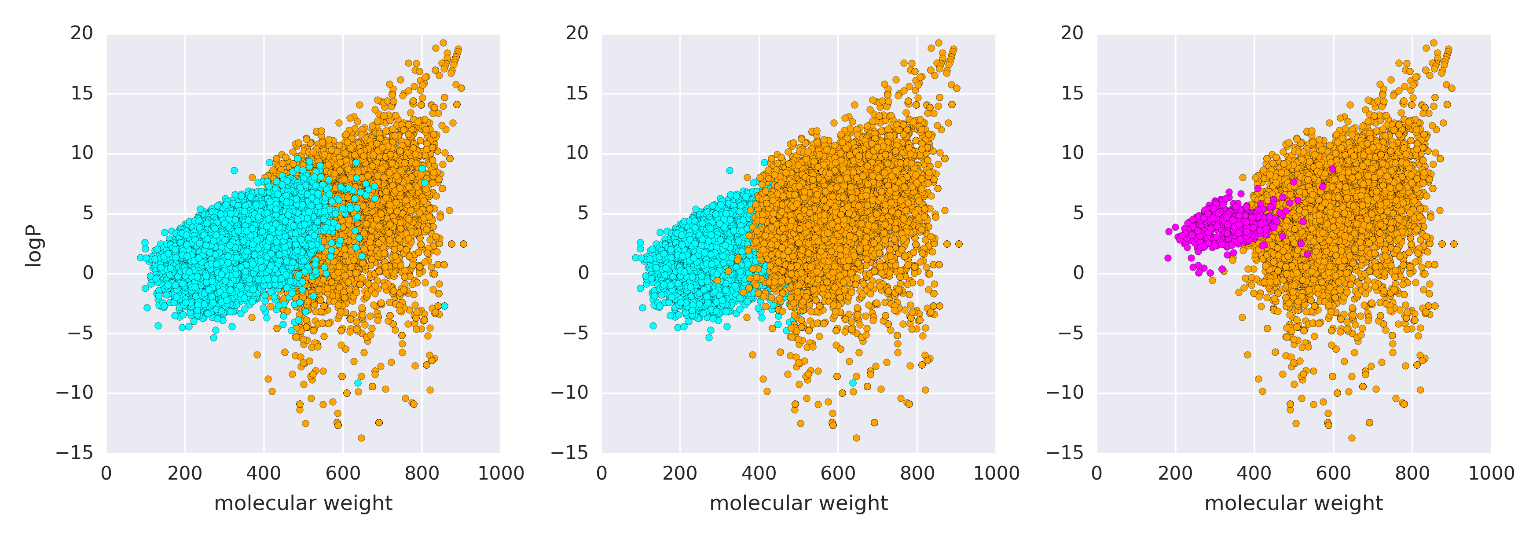
**Fig. S12.** Scatter plot of physicochemical features based on pre-screening for PGH1, a DUD-E target. Each dot represents a compound: cyan dots represent 0.1% of compounds from the ZINC database, orange dots represent the top 0.1% of Spresso-SP results using the method (III) GS_3_ formula, and magenta dots represent active compounds for PGH1 from the DUD-E dataset.

**Fig. S13.** Boxplot representation and average (square dots) of the maximum Tanimoto coefficient between active compounds of target EGFR. The data indicate structural diversity. ZINC, SVM, Glide HTVS, and Spresso represent 0.1% of randomly selected compounds from the ZINC database, the top 0.1% of compounds resulting from SVM prediction, the top 0.1% of compounds resulting from Glide HTVS scoring, and the top 0.1% of compounds returned from Spresso-SP results using method (III) GS_3_ scoring, respectively.

**Fig. S14.** Boxplot representation and average (square dots) of the maximum Tanimoto coefficient between active compounds of target PGH1. The data indicate structural diversity. ZINC, SVM, Glide HTVS, and Spresso represent 0.1% of randomly selected compounds from the ZINC database, the top 0.1% of compounds resulting from SVM prediction, the top 0.1% of compounds resulting from Glide HTVS scoring, and the top 0.1% of compounds returned from Spresso-SP results using method (III) GS_3_ scoring, respectively.

**
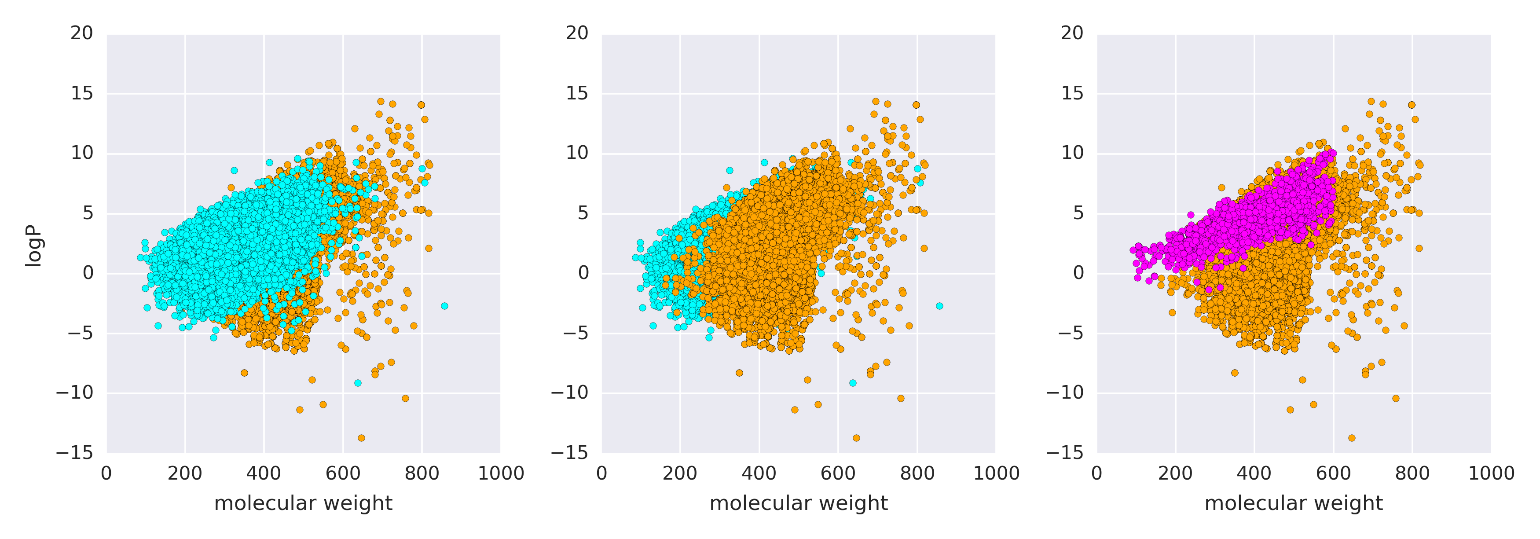
Fig. S15.** Scatter plot of the physicochemical features for ACES. Cyan dots represent 0.1% of compounds from the ZINC database, orange dots represent the top 0.1% of the Spresso-SP results using the GS_3_ formula with cutoff enabled, and the magenta dots represent active compounds for the ACES DUD-E target.

**
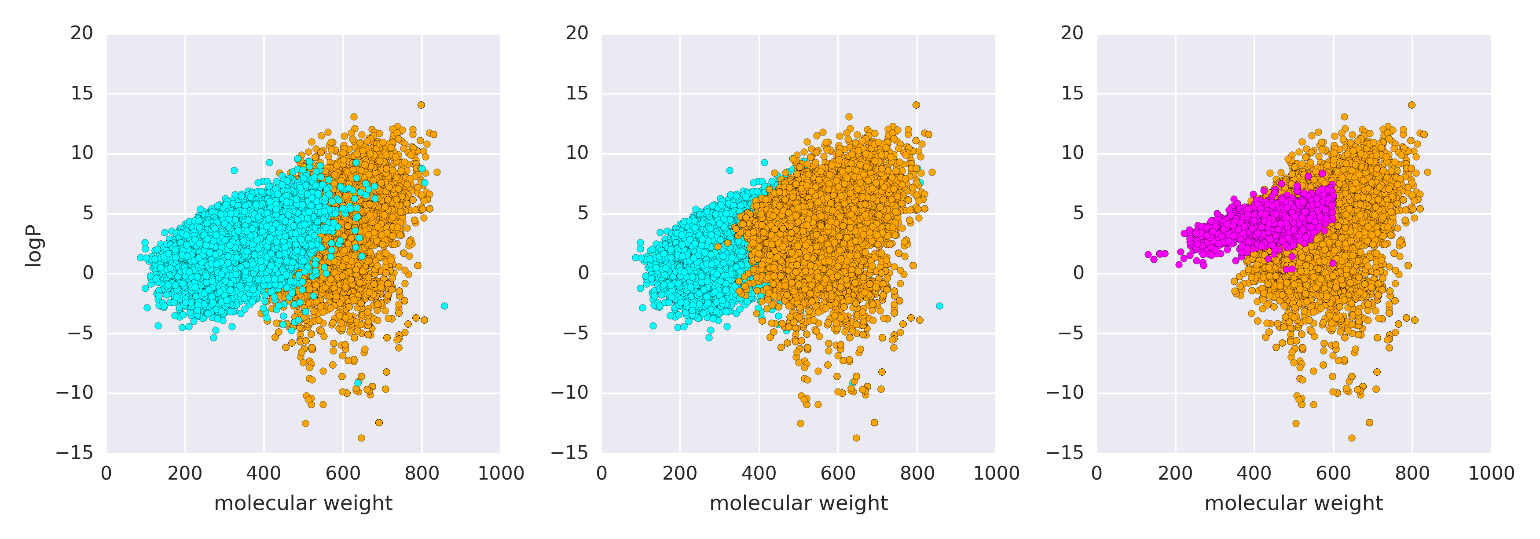
**

**Fig. S16.** Scatter plot of the physicochemical features for EGFR. Cyan dots represent 0.1% of compounds from the ZINC database, orange dots represent the top 0.1% of the Spresso-SP results using the GS_3_ formula with cutoff enabled, and the magenta dots represent active compounds for the EGFR DUD-E target.

**
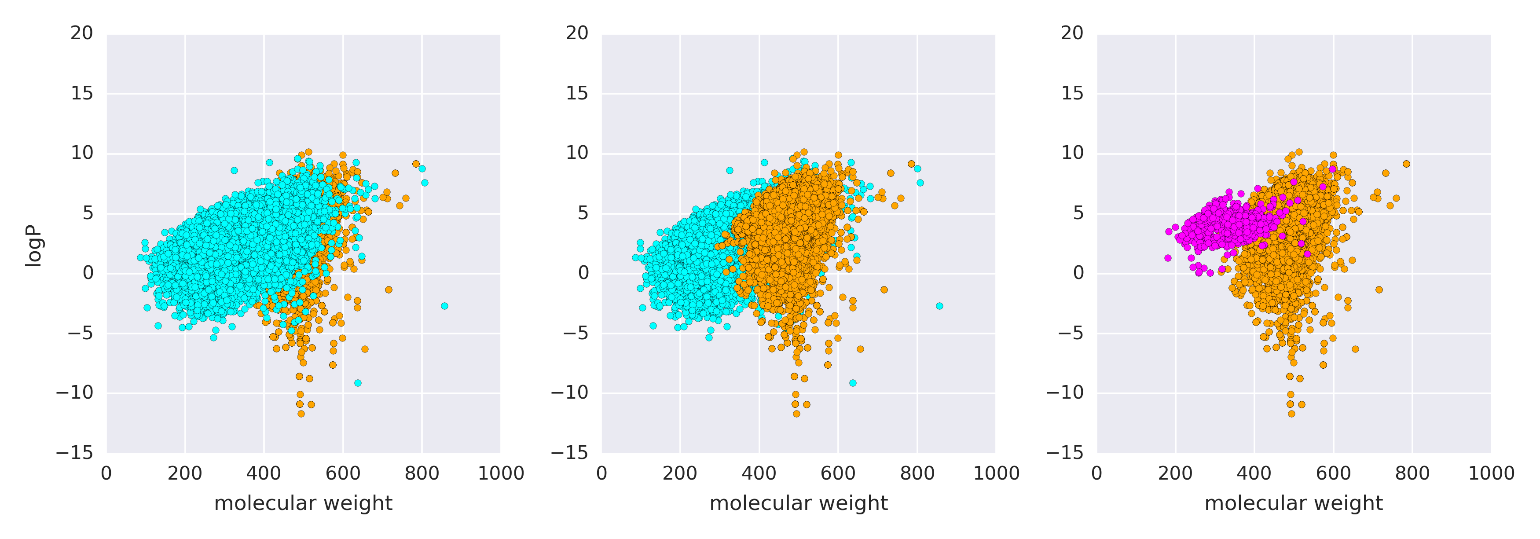
Fig. S17.** Scatter plot of the physicochemical features for PGH1. Cyan dots represent 0.1% of compounds from the ZINC database, orange dots represent the top 0.1% of the Spresso-SP results using the GS_3_ formula with cutoff enabled, and the magenta dots represent active compounds for the PGH1 DUD-E target.

**Fig. S18.** Boxplot representation and average (square dots) of the maximum Tanimoto coefficient between active compounds of target ACES. ZINC, SVM, Glide HTVS, Spresso, and Spresso (w/cutoff) represent 0.1% of randomly selected compounds from the ZINC database, the top 0.1% of compounds from SVM prediction, the top 0.1% compounds from Glide HTVS scoring, the top 0.1% of compounds resulting from Spresso-SP without the cutoff, and the top 0.1% of compounds resulting from Spresso-SP with the cutoff, respectively.

**Fig. S19.** Boxplot representation and average (square dots) of the maximum Tanimoto coefficient between active compounds of target EGFR. ZINC, SVM, Glide HTVS, Spresso, and Spresso (w/cutoff) represent 0.1% of randomly selected compounds from the ZINC database, the top 0.1% of compounds from SVM prediction, the top 0.1% compounds from Glide HTVS scoring, the top 0.1% of compounds resulting from Spresso-SP without the cutoff, and the top 0.1% of compounds resulting from Spresso-SP with the cutoff, respectively.

**Fig. S20.** Boxplot representation and average (square dots) of the maximum Tanimoto coefficient between active compounds of target PGH1. ZINC, SVM, Glide HTVS, Spresso, and Spresso (w/cutoff) represent 0.1% of randomly selected compounds from the ZINC database, the top 0.1% of compounds from SVM prediction, the top 0.1% compounds from Glide HTVS scoring, the top 0.1% of compounds resulting from Spresso-SP without the cutoff, and the top 0.1% of compounds resulting from Spresso-SP with the cutoff, respectively.
